# Identification of structural motifs critical for human G6PC2 function informed by sequence analysis and an AlphaFold2-predicted model

**DOI:** 10.1042/BSR20231851

**Published:** 2024-01-09

**Authors:** Emily M. Hawes, Derek P. Claxton, James K. Oeser, Richard M. O’Brien

**Affiliations:** Department of Molecular Physiology and Biophysics, Vanderbilt University School of Medicine, Nashville, TN 37232, U.S.A.

**Keywords:** glucose metabolism, glucose-6-phosphatase, structure-function

## Abstract

*G6PC2* encodes a glucose-6-phosphatase (G6Pase) catalytic subunit, primarily expressed in pancreatic islet β cells, which modulates the sensitivity of insulin secretion to glucose and thereby regulates fasting blood glucose (FBG). Mutational analyses were conducted to validate an AlphaFold2 (AF2)-predicted structure of human G6PC2 in conjunction with a novel method to solubilize and purify human G6PC2 from a heterologous expression system. These analyses show that residues forming a predicted intramolecular disulfide bond are essential for G6PC2 expression and that residues forming part of a type 2 phosphatidic acid phosphatase (PAP2) motif are critical for enzyme activity. Additional mutagenesis shows that residues forming a predicted substrate cavity modulate enzyme activity and substrate specificity and residues forming a putative cholesterol recognition amino acid consensus (CRAC) motif influence protein expression or enzyme activity. This CRAC motif begins at residue 219, the site of a common *G6PC2* non-synonymous single-nucleotide polymorphism (SNP), rs492594 (Val219Leu), though the functional impact of this SNP is disputed. In microsomal membrane preparations, the L219 variant has greater activity than the V219 variant, but this difference disappears when G6PC2 is purified in detergent micelles. We hypothesize that this was due to a differential association of the two variants with cholesterol. This concept was supported by the observation that the addition of cholesteryl hemi-succinate to the purified enzymes decreased the *V*_max_ of the V219 and L219 variants ∼8-fold and ∼3 fold, respectively. We anticipate that these observations should support the rational development of G6PC2 inhibitors designed to lower FBG.

## Introduction

Glucose-6-phosphatase (G6Pase) catalyzes the hydrolysis of glucose-6-phosphate (G6P) to glucose and free phosphate (Pi) in the endoplasmic reticulum (ER) where it exists as a multi-component enzyme system that includes a membrane-embedded G6Pase catalytic subunit (G6PC) and transporters for G6P, glucose and Pi [1,2]. The G6P transporter brings G6P from the cytosol to the active site of G6PC in the ER lumen, whereas transporters for glucose and Pi return the reaction products to the cytosol [1,2]. There are three members of the *G6PC* gene family, *G6PC1, G6PC2*, and *G6PC3*, which share approximately 35–50% sequence identity but have distinct tissue expression patterns and physiological roles [1]. *G6PC1*, also known as *G6Pase* or *G6PC*, is mainly expressed in the liver, kidney and intestines where it catalyzes the final step in gluconeogenesis and glycogenolysis. *G6PC2*, also known as *IGRP*, is primarily expressed in pancreatic islets. In mice, *G6pc2* is expressed at approximately 20-fold higher levels in β than α cells, whereas in humans *G6PC2* expression is only approximately 5-fold higher in beta cells than alpha cells [3–5]. *G6PC3*, also known as *UGRP*, is ubiquitously expressed with particularly high levels in the kidney, testis, skeletal muscle, and brain [1].

In beta cells, G6PC2 opposes the action of glucokinase, creating a futile substrate cycle that determines the rate of glycolytic flux and, in turn, the sensitivity of glucose-stimulated insulin secretion (GSIS) to glucose [6–9]. This model is supported by experiments showing reduced G6Pase activity [7] and glucose cycling [10] and elevated glycolysis in isolated *G6pc2* knockout (KO) relative to wild-type (WT) islets [8]. In fasted WT and *G6pc2* KO mice, insulin levels are unchanged, but fasting blood glucose (FBG) is reduced in KO mice due to a leftward shift in the GSIS dose–response curve [7,8,11,12]. These observations are consistent with genome wide association and molecular studies that have linked mutations that reduce *G6PC2* expression or activity with reduced FBG levels [13–17].

Because elevated FBG is associated with increased risk for Type 2 diabetes (T2D), cardiovascular associated mortality (CAM), adverse pregnancy outcomes, heart disease, brain atrophy, and several types of cancer [18–31], G6PC2 inhibitors that lower FBG could have multiple therapeutic benefits. Despite these potential benefits of targeting G6PC2, little is understood about its enzymology and how mutations impact its catalytic activity, knowledge gaps that hinder drug discovery efforts. Two recent discoveries − the publication of the AlphaFold2 (AF2) algorithm for the prediction of protein structure [32,33] and the identification of conditions to express and purify the related, catalytically active G6PC1 protein [34] – have opened the door for investigation of the structure–function relationships of G6PC2. The studies described here validate multiple functionally important motifs in G6PC2 as predicted by sequence analysis and an AF2 model.

## Results

### AF2 and amino acid sequence analyses reveal the presence of multiple putative motifs in G6PC2

**Figure 1:**
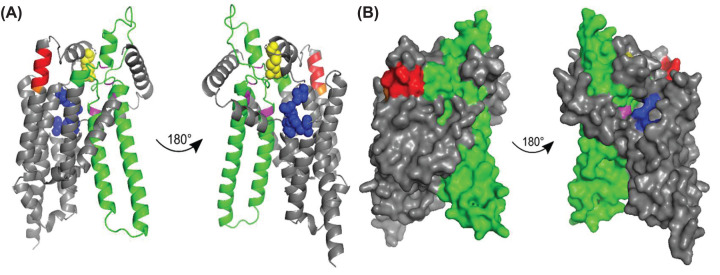
Generation of Predicted G6PC2 Structure Using the AF2 algorithm [32,33], a predicted structure of human G6PC2 was generated and visualized using PyMol [75]. The protein structure is displayed as a cartoon model with certain residues as spheres (**A**) and a space filling model (**B**). In both models, a putative disulfide bond is shown in yellow, a modified phosphatidic acid phosphatase motif [35] is shown in green, a predicted substrate cavity is shown in blue, and a cholesterol recognition amino acid consensus motif [36] is shown in red, with residue 219 in orange.

The predicted AF2 structure of G6PC2 (Figure 1) retains an identical structural fold previously described for G6PC1 (Figure 3 in Reference [33]). Figure 1 shows the protein structure displayed as a cartoon model with certain residues as spheres (Figure 1A) and as a space filling model (Figure 1B). The model predicts a disulfide bond between cysteine residues 105 and 243 and allowed visualization of a substrate cavity leading to the putative active site (Figure 1). Sequence analysis suggests the presence of a modified type 2 phosphatidic acid phosphatase (PAP2) domain [35] and a cholesterol recognition amino acid consensus (CRAC) domain [36] in G6PC2 (Figure 1). The predicted AF2 structure supports the presence of these motifs. Mutational analyses were performed to explore the veracity of these predictions.

### A predicted G6PC2 disulfide bond is critical for G6PC2 protein expression

**Figure 2:**
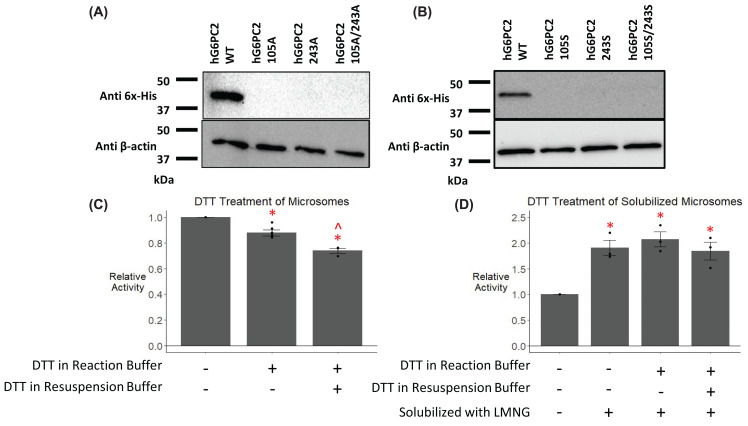
Analysis of the effect of mutation of G6PC2 residues forming a predicted disulfide bond 832/13 cells were transiently transfected with pJPA5 expression vectors encoding either WT or mutated human G6PC2 with a C-terminal His-tag. Following transfection, cells were incubated for 18–20 h in serum-containing media. Cells were subsequently harvested, and G6PC2 expression in whole cell lysates after mutation of the indicated residues to either alanine (**A**) or serine (**B**) was assessed by Western blotting using an anti-6xHis antibody, with equal protein loading confirmed by measurement of actin expression. Representative Western blots are shown (*n*=3). Relative G6Pase activity was determined as described in the 'Experimental Procedures' section and expressed relative to the G6Pase activity of WT G6PC2 in the absence of DTT and LMNG (**C,D**). Reported values are the mean ± SEM (*n*=3–7). *P<0.05 versus no DTT or LMNG, ∧*P*<0.05 versus DTT in reaction buffer as determined by ANOVA with Tukey’s post-hoc test.

The disulfide bond predicted by the AF2 model between cysteine residues 105 and 243 is depicted in Figure 1. Based on a combination of previous studies examining the location of the active site of G6PC1 [35,37,38], conservation between G6PC1 and G6PC2 [39], and the orientation of G6PC2 in the ER [2,40], this disulfide bond is predicted to be located in the ER lumen where there is a relatively high oxidative redox potential, conducive to forming such bonds [41]. We hypothesized that mutation of amino acid 105 and 243 would destabilize the protein if this predicted bond was truly present. To test this hypothesis, we mutated these cysteine residues individually and in tandem to determine the impact on G6PC2 expression. We compared the level of human G6PC2 protein expression driven by plasmid vectors encoding these mutants following transient transfection of the islet-derived 832/13 cell line. Mutation of these residues to alanine, either individually or together, abolished protein expression (Figure 2A). Additionally, we mutated these residues to serine to test the hypothesis that an intramolecular serine-serine hydrogen bond might be able to stabilize the protein. Mutating residues 105 and 243 to serine, either individually or in tandem, failed to restore expression of the protein (Figure 2B). These data indicate that these two cysteine residues are critical for G6PC2 expression, supporting the existence of an intramolecular disulfide bond in G6PC2, as suggested by the AF2 model. However, caveats to this conclusion are presented in the ‘Discussion’ section.

We also investigated the effect of dithiothreitol (DTT) treatment on the G6Pase activity of G6PC2. In microsomal membranes, the addition of 3 mM DTT to either the reaction buffer alone or to both the reaction and resuspension buffers resulted in a modest decrease in G6Pase activity (Figure 2C). After solubilization of G6PC2 using lauryl maltose neopentyl glycol (LMNG), G6Pase activity increased, for reasons that are unclear (Figure 2D). Using solubilized G6PC2, when 3 mM DTT was added to either the reaction buffer alone or to both the reaction and resuspension buffers there was no change in G6Pase activity (Figure 2D). While mutational analyses support the existence of an intramolecular disulfide bond in G6PC2, these studies with DTT suggest that the predicted disulfide bond in G6PC2 appears to be either resistant to cleavage by DTT or not required once the enzyme has been inserted in the ER membrane.

### The predicted PAP2 motif is critical for G6P hydrolysis by G6PC2

**Figure 3:**
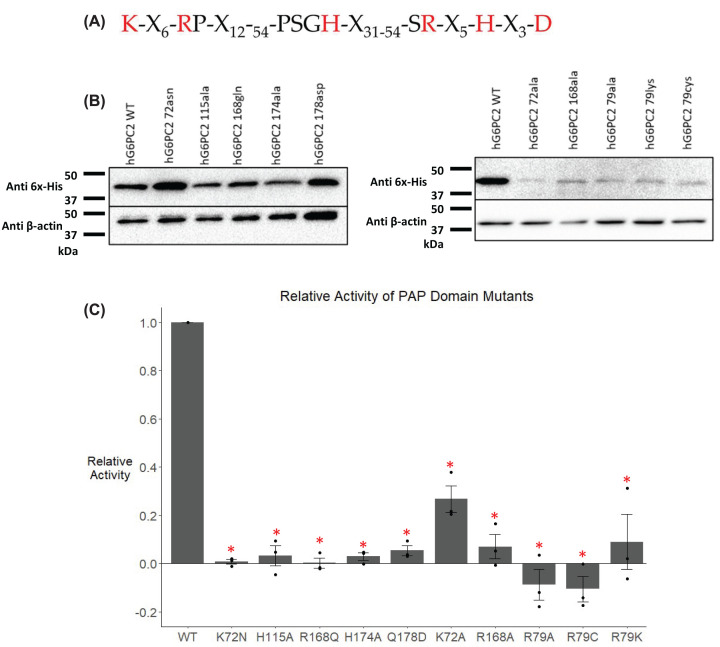
Analysis of the effect of mutation of G6PC2 residues forming a PAP2 motif (**A**) The consensus PAP2 motif sequence; mutations were made to the residues shown in red. 832/13 cells were transiently transfected with pJPA5 expression vectors encoding WT or mutated human G6PC2 with a C-terminal His-tag. Following transfection, cells were incubated for 18–20 h in serum-containing media. Cells were subsequently harvested, and G6PC2 expression in whole cell lysates after mutation of the indicated residues to the amino acid shown was assessed by Western blotting using an anti-6xHis antibody, with equal protein loading confirmed by measurement of actin expression. Representative Western blots are shown (*n*=3–4) (**B**). Relative G6Pase activity (**C**) was determined as described in the ‘Experimental Procedures’ section. Reported values are the mean ± SEM (*n*=3). *P<0.05 compared with WT, as determined by one-way ANOVA, with Dunnett’s post-hoc test.

The AF2 model contains a fold consistent with the presence of a modified type 2 phosphatidic acid phosphatase (PAP2) motif [35,42] as suggested by sequence analyses (Figure 1). The PAP2 motif is comprised of six regions that span from residues 72 to 178 (Figure 3A). The G6PC family PAP2 motif contains a terminal glutamine (found in ∼12% of PAP2 containing proteins) instead of the consensus aspartic acid (found in ∼65% of PAP2 containing proteins) [35,43] (Figure 3A). To test the functional importance of this motif in G6PC2, we mutated one residue in each of the six regions that comprise this motif, including four predicted catalytic residues—Arg79, His115, Arg168, and His174 [37,44] (Figure 3A). Multiple amino acid substitutions at each residue were tested to identify mutants that retained protein expression. Several methods to predict the impact of amino acid substitution on protein function have been reported [45] and a combination of these were used to inform the mutagenesis in these studies. We compared the level of human G6PC2 protein expression driven by plasmid vectors encoding these mutants following transient transfection of the islet-derived 832/13 cell line. This approach was successful except for residue 79 where all mutations tested resulted in reduced protein expression (Figure 3B). To account for these variations in protein expression observed, relative G6Pase activity was determined by dividing enzyme activity by protein expression, both of which were normalized to wild type (WT) levels (Figure 3C). The data show that individual mutation of residues in all six regions of the PAP2 motif resulted in a significant decrease in enzyme activity (Figure 3C). Interestingly, mutation of amino acid 178 from the WT glutamine to the consensus aspartic acid (Figure 3A) resulted in reduced rather than increased enzyme activity (Figure 3C). These data support the AF2 model and indicate that G6PC2 contains a *bona fide* PAP2 motif critical for its hydrolysis of G6P.

### Mutation of the G6PC2 substrate cavity alters G6Pase activity

**Figure 4:**
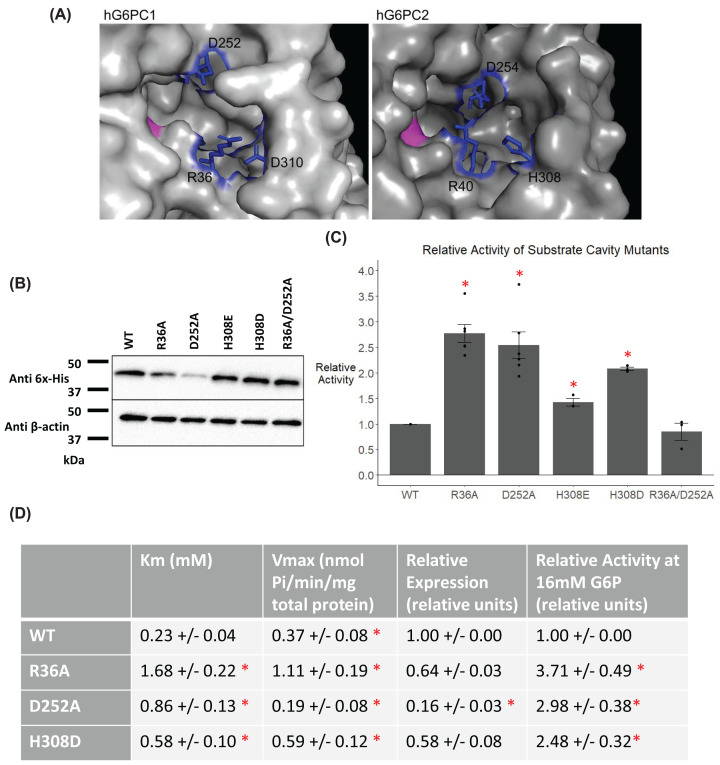
Analysis of the effect of mutation of G6PC2 residues forming a putative substrate cavity (**A**) AF2 predicted structure of human G6PC2 and human G6PC1 [32,33] with key residues in the putative substrate cavity labeled and colored in blue and the putative active site in pink. 832/13 cells were transiently transfected with pJPA5 expression vectors encoding WT or mutated human G6PC2 with a C-terminal His-tag. Following transfection, cells were incubated for 18–20 h in serum-containing media. Cells were subsequently harvested, and G6PC2 expression in whole cell lysates after mutation of the indicated residues to the amino acid shown was assessed by Western blotting using an anti-6xHis antibody, with equal protein loading confirmed by measurement of actin expression. Representative Western blots are shown (*n*=3–5) (**B**). Relative G6Pase activity was determined as described in the ‘Experimental Procedures’ section. Reported values are the mean ± SEM (*n*=3–5) (**C**). (**D**) The kinetics of G6P hydrolysis for WT and mutated human G6PC2 were determined. Reported values are the mean ± SEM (*n*=3–4). *P<0.05 compared with WT as determined by one-way ANOVA with Dunnett’s post-hoc test.

Comparison of the AF2-predicted structures of human G6PC1 and G6PC2 revealed that the putative substrate cavities leading to their active sites have similar features (Figure 4A). In particular, R36 and D252 in human G6PC2, corresponding to R40 and D254 in human G6PC1 are conserved (Figure 4A). We mutated R36 and D252 individually and together to alanine to determine the impact of disrupting these conserved residues on G6PC2 protein expression and enzyme activity. We compared the level of human G6PC2 protein expression driven by plasmid vectors encoding these mutants following transient transfection of the islet-derived 832/13 cell line. The R36A mutant retained WT levels of protein expression whereas the D252A mutant had impaired expression (Figure 4B). To account for these variations in protein expression observed, relative G6Pase activity was determined by dividing enzyme activity by protein expression, both of which were normalized to wild-type (WT) levels (Figure 4C). Individual mutation of either residue, but not both residues together, increased the relative G6Pase activity of G6PC2 (Figure 4C).

The AF2 model predicts that R40 in G6PC1 and R36 in G6PC2 form an ionic interaction in the substrate cavity. In G6PC1, R40 is in close enough proximity to form an interaction with either D254 or D310 (Figure 4A). In G6PC2, the only potential ionic interaction R36 can form is with D252, as residue 308, which corresponds to D310 in G6PC1, is a histidine in G6PC2 (Figure 4A). We hypothesized that the ionic interaction between R40 and D310 in human G6PC1 contributes to the much higher G6Pase activity of G6PC1 relative to G6PC2 [17]. We therefore substituted H308 with aspartic acid in G6PC2 to determine whether it would result in increased activity. We also mutated H308 to glutamic acid to determine whether an acidic residue, rather than aspartic acid specifically, would lead to increased activity. Both mutations had little effect on protein expression (Figure 4B) but, consistent with our hypothesis, both increased relative G6Pase activity (Figure 4C).

The kinetic parameters of these mutants were assessed to determine if any of them altered the apparent affinity for the substrate G6P in addition to relative maximum velocity. The R36A, D252A, and H308D variants increased the *K*m of G6PC2 for G6P approximately 2.5- to 7-fold in addition to increasing the relative activity, suggesting these mutations can release the reaction products quicker than the WT protein (Figure 4D). In combination, these data demonstrate that differences in the putative substrate cavity contribute to the markedly higher G6Pase activity of G6PC1 relative to G6PC2.

### Mutation of the G6PC2 substrate cavity alters substrate specificity

**Figure 5:**
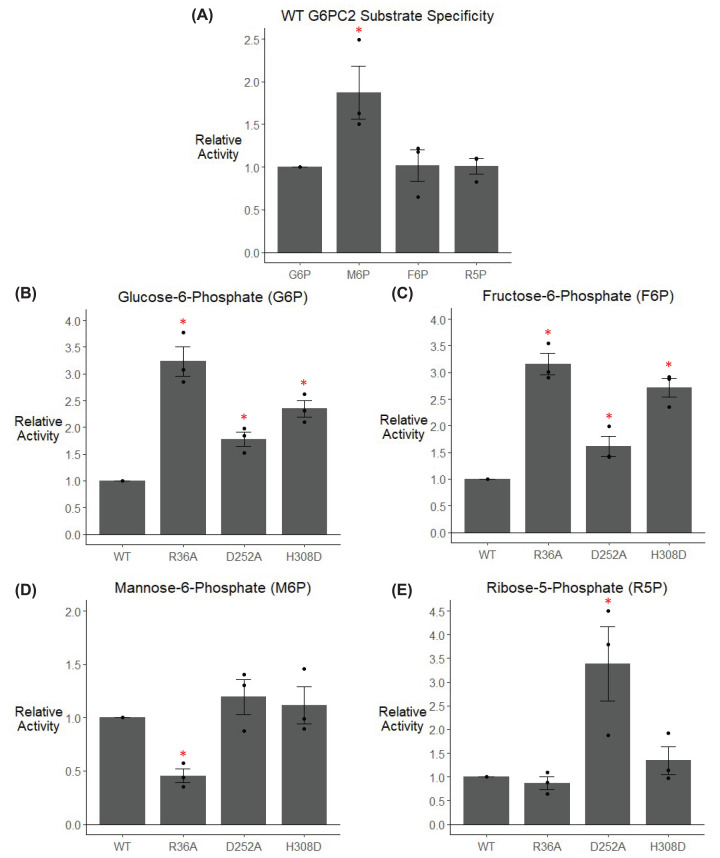
Analysis of the effect of mutation of G6PC2 substrate cavity residues on substrate specificity 832/13 cells were transiently transfected with pJPA5 expression vectors encoding WT or mutated human G6PC2 with a C-terminal His-tag. Following transfection, cells were incubated for 18–20 h in serum-containing media. Cells were subsequently harvested, and G6PC2 expression in whole cell lysates after mutation of the indicated residues to the amino acid shown was assessed by Western blotting using an anti-6xHis antibody, with equal protein loading confirmed by measurement of actin expression. Representative Western blots are shown in Figure 4B. Relative activity was determined as described in the ‘Experimental Procedures’ section. Reported values are the mean ± SEM (*n*=3–6). *P<0.05 versus G6P (**A**) or WT G6PC2 (**B–E**) as determined by one-way ANOVA with Dunnett’s post-hoc test.

In addition to altered substrate affinity, we hypothesized mutations in the putative substrate cavity could affect substrate specificity. Indeed, these mutations had complex effects on the ability of G6PC2 to hydrolyze different sugar phosphate moieties (Figure 5). While WT G6PC2 hydrolyzed G6P, fructose-6-phosphate (F6P), and ribose-5-phosphate (R5P) at similar rates, its hydrolysis of mannose-6-phosphate (M6P) was approximately 2-fold greater (Figure 5A). The R36A, D252A, and H308D variants all increased hydrolysis of G6P and F6P (Figure 5B and C), the R36A variant decreased hydrolysis of M6P (Figure 5D), and the D252A variant increased hydrolysis of R5P (Figure 5E). The complexity of these data suggests that different residues in the substrate cavity play unique roles in the hydrolysis of different sugar phosphate substrates.

### Mutation of a predicted cholesterol binding motif alters maximal activity

**Figure 6:**
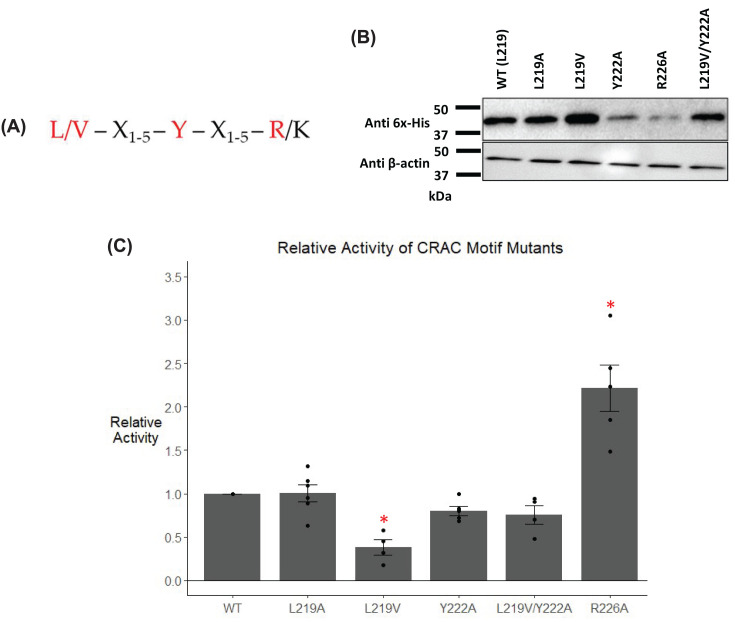
Analysis of the effect of mutation of G6PC2 residues forming a CRAC motif 832/13 cells were transiently transfected with pJPA5 expression vectors encoding WT or mutated human G6PC2 with a C-terminal His-tag. Following transfection, cells were incubated for 18–20 h in serum-containing media. Cells were subsequently harvested, and protein expression assayed by Western blotting as described in the ‘Experimental Procedures’ section. G6PC2 expression was assessed using an anti-6xHis antibody and equal protein loading was confirmed by measurement of actin expression. Consensus CRAC motif with residues mutated highlighted in red (**A**). A representative Western blot is shown (*n*=4–5) (**B**). Relative G6Pase activity was determined as described in the ‘Experimental Procedures’ section. Reported values are the mean ± SEM (*n*=4–5) (**C**). *P<0.05 compared with WT as determined by one-way ANOVA with Dunnett’s post-hoc test.

The AF2 model predicts that the residues comprising a cholesterol recognition amino acid consensus (CRAC) motif [36] in G6PC2 are located on the exterior of the protein (Figure 1). We initially identified this motif through sequence analysis of human G6PC2 (Figure 6A), though the predictive power of this sequence alone is disputed [46–49]. This CRAC motif spans from residue 219 to 226 (Figure 6A). In humans, residue 219 is the site of a common non-synonymous SNP, rs492594 [4]. In the European population, alleles encoding a leucine or valine at residue 219 are in approximately equal abundance [50]. The first residue in the consensus CRAC motif can be either a valine or leucine (Figure 6A), suggesting that both common variants of G6PC2 (L219 and V219) might support cholesterol binding. We hypothesized that mutating residues in this region may alter G6PC2 activity by altering the interaction of G6PC2 with cholesterol. The three critical residues in this motif, located at positions 219, 222, and 226, were mutated to alanine and the effect on G6PC2 expression and G6Pase activity were assessed. In addition, we compared the activity of the V219 and L219 variants and analyzed a L219V/Y222A double mutant. We compared the level of human G6PC2 protein expression driven by plasmid vectors encoding these mutants following transient transfection of the islet-derived 832/13 cell line. We found that G6PC2 expression was similar to WT regardless of the amino acid at residue 219, but mutation of residue 222 or 226 to alanine decreased expression (Figure 6B). To account for these variations in protein expression observed, relative G6Pase activity was determined by dividing enzyme activity by protein expression, both of which were normalized to wild type (WT) levels (Figure 6C). The impact of these mutations on relative G6Pase activity was complex; mutation of residue 219 to valine decreased activity, mutation of residue 226 to alanine increased activity, and all other mutants had no effect on activity (Figure 6C). These data suggest that mutating residues in the CRAC motif can regulate G6PC2 activity, but from these studies it was unclear whether this was due to altered association with cholesterol or due to other altered properties, such as changes in protein conformation.

### Purified G6PC2 L219 and V219 variants exhibit similar activities

**Figure 7:**
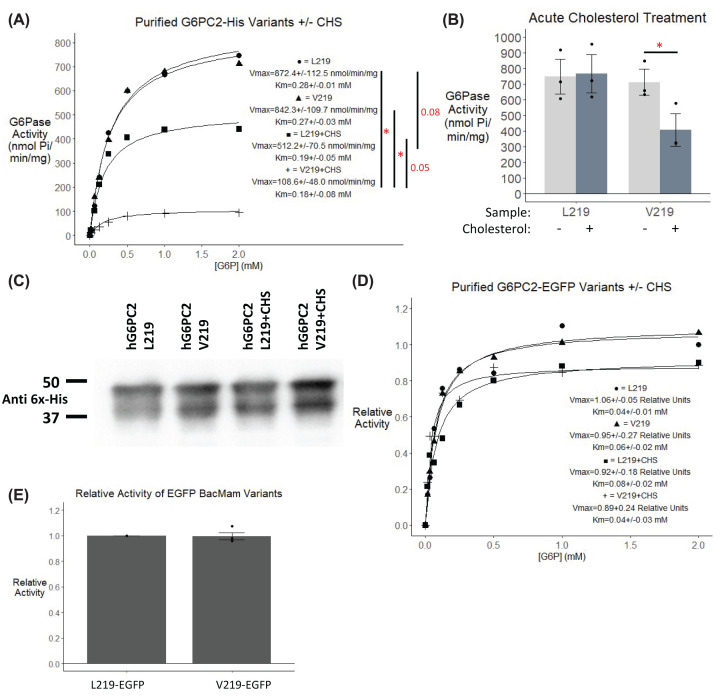
Analysis of the kinetics of human G6PC2 L219 and V219 variants The human G6PC2 L219 or V219 variants with a C-terminal His-tag (**A,B**) or C-terminal EGFP-tag (**D,E**) were expressed using baculovirus in HEK293S cells (**A,B,D**) or using transient transfection in 832/13 cells (**E**) as described in the ‘Experimental Procedures’ section. Cells were then harvested and G6PC2 purified with or without the addition of CHS during solubilization as indicated. Concentration of purified protein was determined using a nanodrop and equal amounts of protein, confirmed by Western blot analysis, were used in each assay. A representative Western blot of His-tagged proteins is shown (*n*=3) (**C**). Representative datasets are shown with the graph line indicating the average kinetic curve as calculated using R (**A,D**). (**A,B**) G6Pase activity was determined as described in the ‘Experimental Procedures’ section. Reported values are the mean ± SEM (*n*=3). *P<0.05 for *V*_max_, as determined by one-way ANVOA with Tukey’s post-hoc test. No statistical differences in *K*_m_ were observed. (**D,E**) Relative G6Pase activity was determined as described in the ‘Experimental Procedures’ section. Reported values are the mean ± SEM (*n*=2–5). No significant differences in *V*_max_ or *K*_m_ were observed, as determined by one-way ANOVA with Tukey’s post-hoc test (**D**) or Student’s *t*-test (**E**).

We hypothesized that the approximately 50% lower activity of the V219 variant relative to the L219 variant in microsomal membranes was due to a differential association of the two variants with cholesterol. To test this hypothesis we modified a recently reported protocol for the overexpression, solubilization and purification of catalytically active G6PC1 [34]. Specifically, instead of heterologous expression in an insect cell host, we expressed G6PC2 in HEK293 cells in suspension via baculovirus transduction. This purification protocol resulted in a 1000-fold higher G6PC2 G6Pase specific activity than observed in microsomal membrane preparations (Figure 7A). After purification of the human G6PC2 L219 and V219 variants in LMNG micelles, we strikingly no longer observed a difference in the activity of the L219 and V219 variants (Figure 7A). We interpreted this observation to reflect the loss of functionally relevant lipids, including cholesterol, following detergent solubilization. When the variants were purified in the presence of cholesterol hemi-succinate (CHS), a cholesterol analog with higher aqueous solubility [51], the *V*_max_ of the V219 variant was reduced ∼90% whereas the *V*_max_ of the L219 variant was reduced only ∼40% (Figure 7A). This observation suggests that our hypothesis was correct and that the loss of endogenous lipid binding explained the loss of a difference in activity between the purified L219 and V219 variants. In addition, the data suggest that interactions of the V219 variant with the cholesterol derivative are distinct from the L219 variant.

In addition to exploring the effects of purifying G6PC2 in the presence of CHS, we investigated the effects of acute cholesterol treatment on the activity of G6PC2 purified in the absence of CHS. Under these conditions, addition of cholesterol decreased the activity of the V219 but not the L219 variant (Figure 7B). The addition of cholesterol had no effect on the activity of either variant after purification in the presence of CHS (data not shown). We ensured these effects were not due to differences in the amount of protein used in the assay by determining the concentration using a nanodrop and further confirming the expression through Western blot analysis (Figure 7C). These data suggest an important role for cholesterol in the regulation of G6PC2 activity.

For optimization of the G6PC2 purification protocol, we generated EGFP-tagged G6PC2 variants to allow easy tracking of protein expression. Interestingly, we observed that when an EGFP-tag was present on the C-terminal end of the L219 and V219 proteins, there was no difference in the *K*_m_ or *V*_max_ of these variants when purified in the presence or absence of CHS (Figure 7D). In addition, in microsomal membrane preparations, the difference in activity observed between the L219 and V219 His-tagged variants (Figure 6C) disappeared with the EGFP-tagged variants (Figure 7E). We hypothesize that these observations are due to the presence of the EGFP-tag changing the ability of the fusion proteins to interact with lipids. This observation is consistent with other papers showing that differences in the C-terminal tag of a protein can influence protein expression, solubility, and/or activity [52–55].

## Discussion

The AF2 algorithm for the prediction of protein structure has allowed us to visualize the 3D structure of human G6PC2 for the first time (Figure 1). The predicted protein structure and sequence analysis identified several key regions of interest within the protein. The data obtained from the studies described here highlight the utility of structural models in identifying regions of importance throughout a protein and demonstrate that multiple regions influence G6PC2 enzyme activity, which could be targeted therapeutically with small molecule inhibitors (Figures 2-7).

Mutation of the predicted disulfide bond in G6PC2 abolished protein expression (Figure 2A) and this could not be restored by replacing these residues with serine residues capable of forming a hydrogen bond (Figure 2B). These data indicate that these two cysteine residues, that are predicted by AF2 to form a disulfide bond, are critical for protein folding and/or protein stability. This is consistent with the observation that mutation of one of the equivalent residues in G6PC1, cysteine residue 109, also results in a significant decrease in protein expression [56]. Interestingly, in assays using isolated microsomal membranes, the addition of DTT to the reaction buffer or the reaction and resuspension buffer had little effect on G6PC2 activity (Figure 2C). Similarly, repeating this analysis with the addition of LMNG to solubilize G6PC2, showed that DTT did not change G6Pase activity (Figure 2D). We hypothesize that this negative result indicates that, once G6PC2 is inserted in the ER membrane, the disulfide bond predicted by AF2 is dispensible for activity. However, other methods, such as mass spectrometry, will be required to definitively determine if this disulfide bond exists.

Multiple residues in the G6PC2 PAP2 motif are critical for enzyme activity (Figure 3). These data fit with previous mutagenesis studies on PAP2 motif containing proteins [35], including studies on G6PC1 showing that mutation of residues in its PAP2 motif result in loss of enzyme activity [35,37,38,44]. Interestingly, mutation of amino acid 178 from glutamine, as found in the G6PC family of proteins, to the aspartic acid found in most PAP2 motif containing proteins (Figure 3A), resulted in reduced, rather than increased enzyme activity (Figure 3C). This suggests a unique evolutionary divergence of the G6PC sub-family of PAP2 motif proteins. Mutating residue 178 from glutamine to aspartic acid does not impact protein expression, which we hypothesize is because these amino acids are similarly sized and should both be capable of forming hydrogen bonds with nearby residues. Instead, due to the internal location of this residue within the protein and its close proximity to the active site residues, we hypothesize that modifying the charge of this residue prevents the enzyme from being able to hydrolyze G6P, possibly through changing the polarity of the active site, since residue 178 is near the phosphate acceptor, H176.

Prior to the publication of the AF2 algorithm, attempts were made to identify residues that explained the marked difference in the G6Pase activity of G6PC1 and G6PC2, but these studies had limited success [57,58]. In contrast, while the accuracy of sidechains projections in AF2 predicted structures is debated [33,59], the predicted structure provided an excellent template for hypothesis generation that we have validated through experimentation. For example, in the AF2-generated structure of human G6PC2, R36 is predicted to interact with D252. In contrast, in human G6PC1 the equivalent residue, R40, is predicted to interact with D310. This putative interaction cannot occur in human G6PC2 as the aspartic acid is replaced with histidine at the equivalent position (H308). We hypothesized that this difference in ionic interactions partially explains why G6PC2 has decreased activity compared with G6PC1. Our mutational analyses support the hypothetical role of such side chain interactions in catalysis as mutation of residue 308 to aspartic or glutamic acid, that would allow an ionic interaction to occur between residues 36 and 308 in G6PC2, increase G6Pase activity (Figure 4). Multiple mechanisms may explain the increase in activity caused by these residue 308 mutations. For instance, charged residues can be used to capture and position substrates in an active site [60]. The benefit of additional charged residues in the substrate cavity may also explain why individual mutation of residues 36 and 252, which would break the predicted ionic interaction and make another charged residue available for interacting with the substrate, increase the G6Pase activity of G6PC2 whereas mutation of both residues does not (Figure 4). Alternatively, studies in G6PC1 have suggested that D254, the residue corresponding to D252 in G6PC2, may directly interact with G6P during hydrolysis and support the formation of a substrate bound state [61]. By promoting a potential ionic interaction between residues 36 and 308 in G6PC2, this may allow D252 to interact with the substrate. While these mutatgenesis studies show that there are residues critical to the activity and expression of G6PC2, we acknowledge that from these studies alone, we can only speculate as to how these mutations are exerting their effects. While advances have been made in using AF2 to predict how mutations will impact protein structure and stability, these techniques are still being optimized [62–64]. Future studies will use molecular dynamics (MD) modeling to investigate how different sugar phosphate moietites fit into the active site of G6PC2 and how mutations to this region impact these interactions.

Our data suggest that cholesterol can modulate the activity of G6PC2 (Figure 7). Additionally, our previous studies with mouse G6PC1 showed that enzyme activity decreased in the presence of cholesterol hemisuccinate [34]. We hypothesize that this regulation by cholesterol is physiologically relevant because, while the concentration of cholesterol in the endoplasmic reticulum is relatively low [65], it matches those used in our studies. While we would expect that the attenuation of G6PC2 activity by cholesterol would result in an increase in GSIS at submaximal glucose concentrations [7], this effect of cholesterol may be masked by its other actions on beta cell function. Indeed, islet cholesterol overload decreases GSIS [66] and reduces plasma insulin levels [67]. While these studies strongly imply that G6PC2 activity is modulated by cholesterol, a key question is whether other lipids also regulate G6PC2 activity. This appears possible because fatty acids [68,69] and the phospholipid composition of liver microsomes [70] impact rat G6PC1 activity. In addition, other PAP domain containing enzymes are also regulated by phospholipids and sphingolipids [71]. Interestingly, the putative CRAC motif found in human G6PC2 is also present in mouse G6PC2 and mouse G6PC1 but is not conserved in human G6PC1. Future studies will explore the potential differential regulation of these enzymes by lipids. Single-particle cryo-transmission electron microscopy (SP-TEM) will also be considered as a future approach to visualize potential G6PC2–lipid interactions which could validate these findings [72].

Overall, these studies provide insight into the molecular mechanisms underlying G6PC2 activity. G6PC2 has been proposed as a drug target for the treatment of patients with elevated FBG levels; the data obtained from these studies will be invaluable in future experiments that seek to design or optimize small molecule inhibitors of G6PC2.

## Experimental procedures

### G6PC expression vector construction

The construction of a plasmid encoding human G6PC2 with a C-terminal His-tag in a modified pJPA5 expression vector was previously described [17].

An EGFP cassette encoding a 5′ thrombin recognition sequence and 3′ His-tag was generated using PCR in conjunction with a CMV EGFP plasmid [34] as the template and the following primers:

Sense strand: 5′-CCG CTCGAG GCTAGC AAT GGC CTG GTG-3′; Xho I and Nhe I restriction enzyme sites, respectively, are underlined.
Anti-sense strand: 5′-CCG CTCGAG TCTAGA TTA GTG ATG GTG-3′; Xho I, Xba I restriction enzyme sites and stop codon, respectively, are underlined. The PCR fragment was digested with Xho I and ligated into Xho I digested pGEM7. The EGFP cassette was then sub-cloned from pGEM7 as a Nhe I - Xho I fragment into the previously described human G6PC2 no Tag pJPA5 MOD plasmid [17] that had been digested with Nhe I and Xho I to generate a plasmid encoding G6PC2 with a C-terminal EGFP-tag.

For baculovirus construction, the human G6PC2 open reading frames with either a C-terminal His-tag or EGFP-tag were isolated from the plasmids described above as EcoR I - Xba I fragments and ligated into the pEG BacMam vector that had been digested with EcoR I and Xba I.

Site-directed mutagenesis using the Quikchange II kit (Agilent Technologies, Santa Clara, CA) was used to change specific codons in human *G6PC2* in the context of the modified pJPA5 and BacMam vectors described above. DNA sequencing was used to verify all codon changes and the absence of secondary mutations in the open reading frame. The open reading frames of all mutants were sub-cloned into a vector that had not previously been used for site-directed mutagenesis to control for the potential presence of secondary mutations in the vector backbone that would not have been identified by sequencing. Plasmid purification was achieved by centrifugation through cesium chloride gradients [73].

### Cell culture

Rat islet-derived 832/13 cells were grown in RPMI medium supplemented with 10% (vol/vol) fetal bovine serum, 0.05 mM β-mercaptoethanol, 100 U/ml penicillin and 100 μg/ml streptomycin. Sf9 insect cells were grown in serum free Sf-900-II SFM media. HEK293S cells, an immortalized human embryonic kidney cell line, were grown in suspension in DMEM medium supplemented with 10% (vol/vol) FBS.

### Protein expression and Western blotting

To determine whether WT and variant human G6PC2 His-tagged proteins were expressed at similar levels, plasmids (3 μg) encoding these proteins were transfected into semi-confluent 832/13 cells using the lipofectamine reagent (InVitrogen, Waltham, MA) and protein expression was then quantitated by Western blotting using actin as an internal control as previously described [74]. We confirmed signal linearity in these western blot analyses with both the anti-β-actin antibodies [17] and anti-His antibodies (data not shown) over a broad range. Normalized expression was determined as the ratio of 6x-His to actin expression obtained with the variants shown as compared to the ratio obtained with WT human G6PC2.

### Isolation of microsomal membranes

Microsomal membranes were isolated from transfected 832/13 cells as described previously [17].

### Measurement of glucose-6-phosphatase (G6Pase) activity *in vitro*

G6Pase activity present in microsomal membranes isolated from transfected 832/13 cells was quantitated as described previously [17]. G6Pase activity in microsomes from mock transfected 832/13 cells was subtracted from G6Pase activity in microsomes from cells transfected with G6PC2 expression vectors. Normalized activity was determined by dividing the G6Pase activity of G6PC2 variants by the G6Pase activity of the WT protein. Relative G6Pase activity was then calculated by dividing normalized activity by normalized expression (determined as described above). When appropriate, hyperbolas were fitted by a Michaelis–Menten model using R.

### DTT treatment of microsomal membranes

For the analysis of the effect of DTT, DTT was added to the G6Pase reaction buffer and/or microsomal membrane resuspension buffer to a final concentration of 3 mM. Where indicated, the microsomal membrane resuspension buffer included a final concentration of 0.5% LMNG to solubilize G6PC2. For solubilization, the mixture was nutated at 4 C for 1 h before being centrifuged for 30 min at 250,000 rcf to remove insoluble protein. The LMNG concentration was maintained at 0.5% in the G6Pase reaction.

### Recombinant baculovirus production and HEK cell infection

pBacMam plasmids encoding the L219 and V219 G6PC2 His-tagged or EGFP-tagged variants were transformed into DH10Bac competent cells for site-specific transposition of the expression cassette into the bacmid vector. Baculoviruses were generated as previously described [34]. Briefly, isolated bacmid was transfected into adherent Sf9 cells using Cellfectin II. Transfected cells were then incubated for 5 days at 27°C; media containing the P1 baculovirus was then harvested and filtered. To amplify the virus, the P1 virus was diluted 1000-fold into a Sf9 cell suspension growing in serum free Sf-900-II SFM media (Gibco) and incubated for 4 days at 27°C while shaking at 130 rpm. The cells were removed by centrifugation, and the supernatant containing P2 baculovirus was filter sterilized. P2 viral titer was determined; the average P2 viral titer used for experiments was 3 × 10^7^ pfu/ml. P1 and P2 baculovirus preparations were supplemented with 2% FBS and stored at 4°C, protected from light. HEK cells at a density of ∼2 million cells/ml were infected with 10% vol/vol P2 virus. Cells were incubated with shaking for 20–24 h at 37°C. After 20–24 h, sodium butyrate was added to a final concentration of 5 mM. The cells were then shaken for an additional 42–48 h at 30°C (total shaking time 66–72 h) before harvesting.

### Protein solubilization and purification

G6PC2 was purified from HEK cells by adapting a protocol previously designed for the isolation of G6PC1 from Sf9 cells [34]. Briefly, baculovirus-infected HEK cells were pelleted by centrifugation for 15 min at 2800 rcf. The supernatant was removed, and the pellet resuspended in 50 ml lysis buffer (50 mM Tris-HCl pH 8, 50 mM NaCl, 0.5 mM EDTA, 10% glycerol). The solution was then homogenized using a glass homogenizer and transferred to a beaker. The solution was stirred continuously and protease inhibitors (benzamidine (1:500), leupeptin, pepstatin, chymostatin, and PMSF (1:1000)) were added. Cells were then lysed, while still being stirred, by sonication for 8 min (10 s on, 10 s off, 40% amplitude with a minute break after 4 min). Cell debris was removed by centrifugation for 20 min at 10,000 rcf. The supernatant was then subjected to a high-speed spin (200,000 rcf for 1.5 h) to isolate microsomal membranes. The microsomal membranes were kept on ice overnight before resuspension in a buffer (50 mM Tris-HCl pH 8, 100 mM NaCl, 10% glycerol) containing LMNG (0.5%) or LMNG plus CHS (0.05%) at a final concentration of 80 mg/ml membrane protein. The mixture was then stirred on ice for 1 h before being centrifuged for 30 min at 250,000 rcf. The supernatant, containing the membrane fraction that had been resuspended in detergent, was filtered, imidazole was added to a concentration of 25 mM, and added to nickel resin pre-equilibrated with the resuspension buffer. This solution was nutated for 2 h at 4°C. The solution was then poured into a column and the resin washed with five column volumes of washing buffer (resuspension buffer with 50 mM imidazole). The protein was then eluted from the resin with elution buffer (resuspension buffer with 300 mM imidazole). Eluted protein was then injected onto a size exclusion column that had been equilibrated with gel filtration buffer (50 mM Tris pH 7.5, 150 mM NaCl, 10% glycerol). The washing, elution, and gel filtration buffers also contained 0.2% LMNG with or without 0.02% CHS. Protein concentration in the peak protein fraction was determined using a nanodrop at an absorbance of A280 and corrected for the extinction coefficient (2.1).

### Statistical analysis

Enzyme activity data were analyzed using either the Student’s *t*-test: two sample, two-sided test assuming equal variance or using analysis of variants (ANOVA) test with the Tukey’s Honest Significant Difference or Dunnett’s Multiple Comparison post-hoc test, as stated. Briefly, a Tukey’s post-hoc test was used when all samples were compared and a Dunnett’s post-hoc test was used when samples were compared only with the WT. *P-*values are indicated.

## Data Availability

The data needed to evaluate this work are all included in the manuscript and are available upon request from R. O’B. (richard.obrien@vanderbilt.edu)

## Abbreviations

AF2: AlphaFold2
CAM: cardiovascular associated mortality
CHS: cholesterol hemi-succinate
CRAC: cholesterol recognition amino acid consensus
DTT: dithiothreitol
ER: endoplasmic reticulum
F6P: fructose-6-phosphate
FBG: fasting blood glucose
G6P: glucose-6-phosphate
G6Pase: glucose-6-phosphatase
G6PC: glucose-6-phosphatase catalytic subunit
G6PC1: glucose-6-phosphatase catalytic subunit 1
G6PC2: glucose-6-phosphatase catalytic subunit 2
GSIS: glucose-stimulated insulin secretion
LMNG: lauryl maltose neopentyl glycol
M6P: mannose-6-phosphate
PAP2: type 2 phosphatidic acid phosphatase
Pi: free phosphate
R5P: ribose-5-phosphate
SNP: single-nucleotide polymorphism
T2D: Type 2 diabetes
